# Autophagy-Related Proteins Target Ubiquitin-Free Mycobacterial Compartment to Promote Killing in Macrophages

**DOI:** 10.3389/fcimb.2016.00053

**Published:** 2016-05-11

**Authors:** Aïcha Bah, Camille Lacarrière, Isabelle Vergne

**Affiliations:** Tuberculosis and Infection Biology, Institut de Pharmacologie et de Biologie Structurale, UMR 5089 Centre National de la Recherche Scientifique - Université de ToulouseToulouse, France

**Keywords:** autophagy, mycobacterium, macrophage, ubiquitin, toll-like receptor, phagosome, innate immunity

## Abstract

Autophagy is a lysosomal degradative process that plays essential functions in innate immunity, particularly, in the clearance of intracellular bacteria such as *Mycobacterium tuberculosis*. The molecular mechanisms involved in autophagy activation and targeting of mycobacteria, in innate immune responses of macrophages, are only partially characterized. Autophagy targets pathogenic *M. tuberculosis* via a cytosolic DNA recognition- and an ubiquitin-dependent pathway. In this report, we show that non-pathogenic *M. smegmatis* induces a robust autophagic response in THP-1 macrophages with an up regulation of several autophagy-related genes. Autophagy activation relies in part on recognition of mycobacteria by Toll-like receptor 2 (TLR2). Notably, LC3 targeting of *M. smegmatis* does not rely on membrane damage, ubiquitination, or autophagy receptor recruitment. Lastly, *M. smegmatis* promotes recruitment of several autophagy proteins, which are required for mycobacterial killing. In conclusion, our study uncovered an alternative autophagic pathway triggered by mycobacteria which involves cell surface recognition but not bacterial ubiquitination.

## Introduction

Macroautophagy, hereafter referred to as autophagy, is a eukaryotic lysosomal degradative process involved in removal and recycling of cytoplasmic components. In addition to its ubiquitous role in cellular homeostasis, autophagy plays major functions in immune defenses against intracellular bacteria, for instance, as effector of pattern recognition receptors (PPRs) and regulator of inflammation or in favoring antigen presentation and bacterial clearance (Deretic et al., 2013).

Autophagy is carried out through the coordinated action of more than 30 autophagy-related proteins (Atgs), several of them, organized in different functional complexes (Lamb et al., 2013). The process is initiated by activation of Ulk1/Atg13/FIP200/Atg101 and Beclin-1/hVps34/Atg14 complexes which result in the formation of an isolation membrane. This membrane elongates to engulf cytoplasmic cargo and then fuses with itself to form a double-membrane bound organelle called autophagosome. At this stage, two ubiquitin-like conjugation systems are required: (i) the covalent linkage of Atg12 with Atg5 in complex with Atg16L1; (ii) LC3 lipidation with phosphatidylethanolamine. Once the autophagosome formed, Atg5-12/Atg16L1 protein complex is released while LC3 remains on the autophagosomal inner membrane. Autophagosome ultimately undergoes fusion with lysosomes leading to the degradation of sequestered cargo and LC3-decorated inner membrane. Several proteins participate in autophagosome-lysosome fusion event including the small GTPase Rab7, UV radiation resistance-associated gene protein (UVRAG), and the t-SNARE syntaxin 17 (STX17; Gutierrez et al., 2004b; Liang et al., 2008; Itakura et al., 2012; Takats et al., 2013). Overall, the autophagic process is highly regulated through multiple post-translational modifications of Atgs, transcriptional and post-transcriptional reprogramming triggered by a wide array of signaling pathways (Ravikumar et al., 2010; McEwan and Dikic, 2011; Pietrocola et al., 2013; Fullgrabe et al., 2014).

The molecular mechanisms governing autophagy activation upon bacterial infection are far from being completely understood as they seem to be influenced by numerous factors such as bacterial species, its virulence and its trafficking inside the host cell (Pareja and Colombo, 2013; Huang and Brumell, 2014; Shibutani and Yoshimori, 2014). For instance, intracellular bacteria such as *Shigella, Listeria*, and *Salmonella* are able to damage their vacuolar membrane which triggers a cascade of events leading to autophagy activation, through a mTOR pathway, and selective capture of the bacteria (Tattoli et al., 2012). Selective targeting of *Salmonella* relies on bacterial ubiquitination and recruitment of ubiquitin-binding autophagy receptors such as p62, ndp52, and optineurin (Gomes and Dikic, 2014). These adaptors contain a LC3-interacting region (LIR) that enables recruitment of LC3 and consequently capture of the bacteria. Importantly, a non-canonical autophagic pathway, called LC3-associated phagocytosis (LAP), can also bring about LC3 recruitment to intracellular bacterial compartment (Lai and Devenish, 2012; Mehta et al., 2014). This pathway, initiated after engagement of some receptors located on the cell surface, including TLR2 and TLR4, promotes LC3 conjugation directly onto the phagosomal membrane via an ULK1-independent mechanism.

Mycobacteria are a large family of bacteria which are characterized by a cell envelope rich in unusual lipids and glycoconjugates with potent immunomodulatory properties (Neyrolles and Guilhot, 2011; Vergne et al., 2014). Even though most of mycobacteria are non-pathogenic, a couple of serious human pathogens belong to this family such as *Mycobacterium tuberculosis* and *M. leprae*, etiologic agents of Tuberculosis and Leprosy, respectively. One of the more prominent features of mycobacterial pathogenicity is their ability to survive and replicate in macrophages (Russell, 2011). Pioneering research has shown that autophagy activation plays an important role in controlling mycobacteria intracellular growth in macrophages (Gutierrez et al., 2004a; Yuk et al., 2009; Watson et al., 2012). The molecular mechanisms involved in activation of autophagy in innate immune responses to mycobacterial infection are just starting to be unraveled. In macrophages, a subpopulation of intracellular *M. tuberculosis* is targeted by autophagy via a mechanism that depends on ubiquitination of the bacteria or its compartment and recruitment of autophagy receptors p62 and ndp52 (Watson et al., 2012). A few hours following phagocytosis, *M. tuberculosis* Esx-1 secretion system damages phagosomal membrane, and, as a result, exposes mycobacterial extracellular DNA to the cyclic GMP-AMP synthase (cGAS)/STING-dependent cytosolic DNA sensing pathway which triggers bacterial ubiquitination (Watson et al., 2012, 2015). Interestingly, mycobacterial recognition by toll-like receptors (TLRs) appears to participate in this autophagic pathway via expression of DNA damage-regulated autophagy modulator DRAM1 (van der Vaart et al., 2014). Esx-1 is also implicated in LC3 targeting of *M. marinum*, however, ubiquitin does not seem to be required for this process (Lerena and Colombo, 2011). Importantly, several studies indicate that pathogenic mycobacteria can limit autophagic response in macrophages (Espert et al., 2015). Indeed, a report shows that non-pathogenic mycobacteria, such as *M. smegmatis* which is killed by macrophages, induce a stronger autophagic response than *M. tuberculosis*, suggesting possible additional mechanism for autophagy activation (Gutierrez et al., 2008; Zullo and Lee, 2012). *M. smegmatis* is a model of non-pathogenic mycobacteria often used to study host immune defense mechanisms during mycobacterial infection (Astarie-Dequeker et al., 1999; Yadav and Schorey, 2006; Alonso et al., 2007; Jordao et al., 2008; Rajaram et al., 2011). More recently, *M. smegmatis* was successfully used as a vaccinal vector (Zhang et al., 2010; Lu et al., 2011; Sweeney et al., 2011). Thus, to advance our understanding of mycobacteria-induced autophagy, we set out to decipher the autophagic response to *M. smegmatis* infection in macrophages and its role in infection. Our study shows that *M. smegmatis* activates autophagy via TLR2 engagement. In addition, autophagy machinery targets *M. smegmatis* and mediates its killing. Importantly, bacterial ubiquitination and autophagy receptors p62 and ndp52 are not implicated in that process.

## Materials and methods

### Reagents

Diphenyleneiodonium chloride (DPI), Dimethylsulfoxide (DMSO), and Earle's balanced salt solution (EBSS) were purchased from Sigma. Bafilomycin A1 was purchased from Santa cruz. The following rabbit antibodies were used: ULK1 (Cell Signaling), Atg13 (E1Y9V, Cell Signaling), Atg16L1 (Thermo Scientific, MBL), Beclin-1 (Santa Cruz), CD63 (Santa Cruz), LC3 (Sigma, MBL), ndp52 (Abcam). The following mouse antibodies were used: β-actin (Abcam, Santa Cruz), IgG1 K isotype (eBioscience), Galectin-3 (BD Pharmingen), p62 (BD Transduction Laboratories), TLR2 (Invivogen), ubiquitin (FK2, Enzo Life Sciences).

### Macrophage and bacteria culture, and infection

Human monocytic THP-1 cells (ATCC TIB-202T) were cultured in complete RPMI 1640 medium (THP-1 medium) (Gibco) containing 10% heat inactivated fetal bovine serum (FBS), 2 mM L-Glutamine, 1 mM sodium pyruvate, and 1% MEM non-essential amino-acids. THP-1 monocytes were differentiated into macrophages with 20 ng/ml phorbol 12-myristate 13-acetate (PMA, Fisher bioreagents) for 24 h at 37°C, 5% CO_2_. PMA was washed away and cells were rested for 1 h in THP-1 medium before infection.

*M. smegmatis* mc^2^155 wild-type (700084) was from ATCC. *M. smegmatis* Δ*pmmB* mutant was obtained from J. Nigou (IPBS, Toulouse; Krishna et al., 2011). Mycobacteria were grown in Middlebrook 7H9 broth (BD Difco) containing 10% albumin-dextrose-catalase (ADC) (BD Difco) and 0.5% glycerol at 37°C. Kanamycin (Euromedex) was added at 40 μg/ml to *M. smegmatis* Δ*pmmB* culture. GFP-expressing *M. tuberculosis* H37Rv Pasteur and *M. smegmatis* were kindly provided by C. Astarie-Dequeker and A.Peixoto (IPBS, Toulouse), respectively. Kanamycin was added at 50 μg/ml to GFP-expressing *M. smegmatis* culture and hygromycin (Invitrogen) at 50 μg/ml to GFP-expressing *M. tuberculosis* culture.

For infection, mycobacteria were washed with phosphate buffered saline (PBS), disaggregated by shaking vigorously for 30 s with glass beads (4 mm diameter) and resuspended in THP-1 medium. Macrophages were infected with bacteria at indicated multiplicity of infection (MOI) in THP-1 medium at 37°C, 5% CO2. After infection, extracellular bacteria were removed by several washes and by killing with 100 μg/ml gentamycin (Sigma) for overnight post-infection incubation.

### Western immunoblotting

THP-1 cells were seeded and differentiated in 25 cm^2^ flasks. After infection and/or treatment as indicated in figure legends, cells were lysed with a Radio-Immunoprecipitation Assay (RIPA) buffer containing protease and phosphatase inhibitor cocktail. Protein concentration was determined using bicinchoninic acid assay (Interchim Uptima) and 40 μg of proteins were subjected to SDS-polyacrylamide gel electrophoresis (4–15% gradient) using Laemmli sample buffer containing β-mercaptoethanol and a Tris/glycine buffer system (BioRad). After electrophoresis, proteins were transferred to a nitrocellulose transfer membrane. Blots were blocked with 5% dried milk in PBS, incubated with primary antibodies and then with the corresponding horseradish peroxidase-conjugated secondary antibody (Thermo Scientific). Staining was detected with SuperSignal West Pico Chemiluminescent Substrate (Thermo Scientific) and immunostained proteins were visualized on lumi-film chemiluminescent detection film (Roche). When necessary, blots were stripped with restore WesternBlot stripping buffer (Thermo Scientific).

### Immunofluorescence, lysotracker, and DQ red BSA assays

THP-1 cells were seeded and differentiated on glass coverslips into 24-well plates. For immunofluorescence and Lysotracker Red experiments, mycobacteria were labeled with Alexa 488 succinimidyl ester (Invitrogen) as described previously (Kyei et al., 2006). Phagocytosis was synchronized by gentle centrifugation of mycobacteria onto THP-1 cells for 5 min. After infection for 30 min, macrophages were washed and incubated as indicated. Cells were then fixed with 2% paraformaldehyde (Electron Microscopy Sciences) for 10 min followed by membrane permeabilization using 0.1% Triton X100 for 5 min. After blocking with 4% BSA, 2% goat serum in PBS, permeabilized cells were incubated with primary followed by secondary Alexa 568–conjugated antibody or Alexa 647-conjugated antibody (Invitrogen). For lysotracker Red (LTR) experiment, cells were incubated for 2 h post-infection with LTR (Invitrogen) at 1 μM followed by several washes and fixation. For DQ Red BSA assay, cells were preincubated for 2 h prior infection with DQ Red BSA (Invitrogen) at 10 μg/ml, washed and then infected with *M. smegmatis*.

The coverslips were mounted onto glass slides with fluorescent mounting medium (Dako) and were analyzed on a Zeiss LSM510 or an Olympus FV1000 confocal microscopes. Around 35 random images were taken per condition per experiment which corresponds to a total of more than 100 mycobacteria from three independent experiments. Images were processed using LSM510 or image J software. Mycobacteria were considered positive when more than 50% of bacterial surface were colocalizing with the studied marker.

### Quantitative real-time reverse transcriptase- polymerase chain reaction (RT-PCR)

Total RNA was extracted from THP-1 macrophages using RNAeasy kit (Qiagen) and was reverse transcribed into cDNA using the SuperScript III First-Strand Synthesis System (Invitrogen) and oligo-dT, according to the manufacturer's protocols. Quantitative PCR was performed in duplicate for each studied gene using a CFX96 Real Time PCR System (Biorad). KAPA SYBR Fast Universal Ready mix Kit containing SYBR Green dye was used for amplification following the manufacturer's instructions (Kapa Biosystems). Quantification was achieved using the CFX manager software 2.1 (Biorad). Gene expression was expressed as relative copy number (RCN). RCN was calculated using with the following equation: RCN = 2^−ΔCt^ × 100 where ΔCt corresponds to average Ct values of each studied gene subtracted from average Ct of GADPH (housekeeping gene). RCN represents gene expression as number of copies relative to the 100 copies of housekeeping gene.

Oligonucleotide primers were designed in PRIMER3 PLUS and analyzed in OligoAnalyser online softwares. The following set of validated primers, synthesized and purchased from Sigma, were used: Atg5: GCAAGCCAGACAGGAAAAAG (F), GACCTT CAGTGGTCCGGTAA (R); Atg14: TCCATTTTCCCATCCAGTTC (F), AAGTCAGTC TCCACCACCAA (R); Beclin-1: TGAGGGATGGAAGGGTCTAA (F), TGGGCTGTG GTAAGTAATGG (R); LC3B: AACGGGCTGTGTGAGAAAAC (F), AGTGAG GACTTTGGGTGTGG (R); STX17: CCAGCCAAACTGACAAGAAA (F), ACA CCCCAGCAAACAACAA (R); hVps34: ATGGAAGCCGATGGATGTAG (F), CCTCACAGTTGGGTTGGTG (R); ULK1: CACACGCCACATAACAGACA (F), CCCCACAAGGTGAGAATAAAG (R); UVRAG: GAGTTGGGGTGTCTG GTAGG (F), AATCTGAATGCGGGAATGAC (R). All data were normalized to GAPDH: CCATGTTCGTCATGGGTGTG (F); GGTGCTAAGCAGTTGGTGGTG (R).

### Knockdown experiments

THP-1 cells were transfected with siRNA (final concentration 110 nM) using HiPerFect transfection reagent (Qiagen) according to the manufacturer's protocol in presence of 20 ng/ml PMA for 24 h. ULK1, BECN1 (Beclin-1), and ATG16L1 knock-downs were achieved by using siGENOME SMARTpool reagent (Dharmacon) specific for *Homo Sapiens*. All effects of ULK1, BECN1, and ATG16L1 siRNAs were compared with siCONTROL Nontargeting siRNA pool (Dharmacon) which is recommended by the manufacturer as siRNA control and used by others in autophagy studies (Lerner et al., 2016).

### Mycobacterial survival

THP-1 were infected with *M. smegmatis* at MOI 2. After 30 min infection, cells were washed, incubated in THP-1 media for 30 min and, then, either directly lysed with cold water for uptake measurement or lysed after 40 h incubation in presence of 100 μg/ml of gentamycin to kill extracellular bacteria. Serial dilutions of bacteria were plated on Middlebrook 7H11 agar plates with OADC in duplicate and colony-forming unit (CFU) were counted after 3 days of incubation. For each experiment the average of the duplicate was determined.

### Statistical analysis

Data are shown as mean ± SEM from at least three independent experiments. Statistical analysis was performed with Graphpad Prism version 5 software using Student's two-tailed *t*-test. Differences were considered significant when *p*-value was inferior to 0.05.

## Results

### *M. smegmatis* induces autophagy in THP-1 macrophages

**Figure 1:**
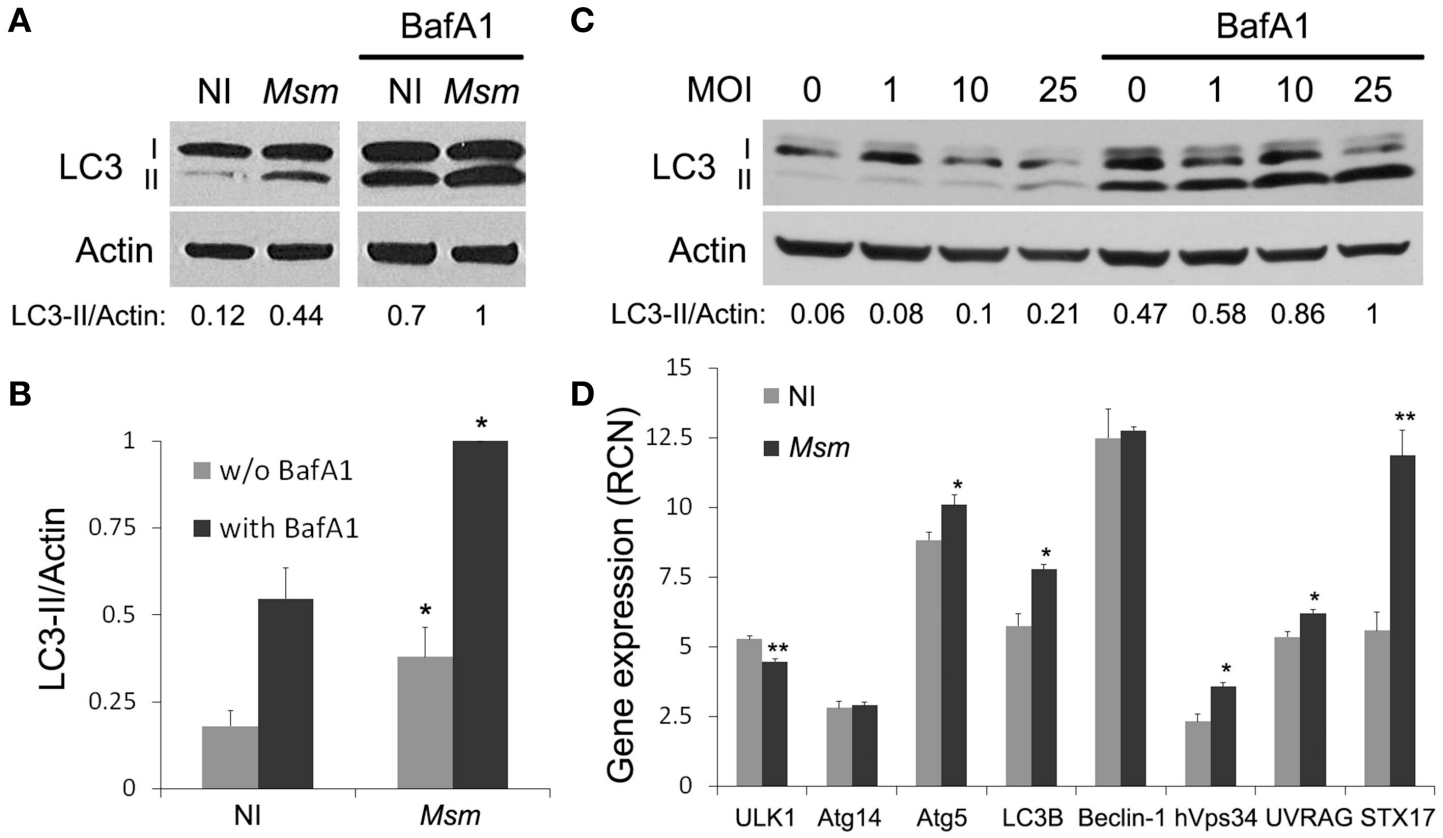
*****M. smegmatis*** induces autophagy in THP-1 macrophages. (A,B)** LC3 immunoblotting of THP-1 macrophages infected (*Msm*) or not (NI) with *M. smegmatis*. Differentiated THP-1 cells were incubated with *M. smegmatis* at multiplicity of infection (MOI) 25 for 2 h, washed, and then incubated for 1 h and 30 min with 100 nM Bafilomycin A1 (with BafA1) or DMSO control (w/o BafA1). Cells were lysed and analyzed by immunoblotting with anti-LC3 or anti-actin. Actin was used as a loading control. Densitometric LC3-II/actin ratios are shown underneath the blot **(A). (B)** Quantification of LC3-II/actin ratios. Ratios were normalized to ratio of infected cells with BafA1. Data, mean ± SEM (*n* = 6 independent experiments), ^*^P < 0.05 (paired *t*-test, infected vs. non-infected cells). **(C)** LC3 immunoblotting of THP-1 macrophages infected with *M. smegmatis (M. sm)* for 24 h at different MOI (0–25). Differentiated THP-1 cells were incubated with *M. smegmatis* for 2 h, washed, and then incubated for 24 h. Cells were treated with 100 nM Bafilomycin A1 or DMSO control during the last 2 h. Cells were lysed and analyzed by immunoblotting with anti-LC3 or anti-actin. Densitometric LC3-II/actin ratios are shown underneath the blot. **(D)** Quantitative real-time PCR analysis of autophagy-related gene transcripts upon *M. smegmatis* infection. Differentiated THP-1 cells were incubated (*Msm*) or not (NI) with *M. smegmatis* at MOI 10 for 2 h, washed and incubated for 6 h. After lysis, RNA was extracted and RT-PCR analysis was performed for selected autophagy pathway genes. Data are expressed as relative copy numbers (RCN) to GADPH (house-keeping gene). Data, mean ± SEM (*n* = 3 independent experiments), ^*^P < 0.05, ^**^P < 0.01 (unpaired *t*-test).

So far, autophagic response to non-pathogenic mycobacteria, such as *M. smegmatis*, has been studied in murine RAW264.7 macrophages (Zullo and Lee, 2012). To determine whether this response is conserved in human cells, we investigated autophagy in another cellular model commonly used to study mycobacteria-macrophage interaction, THP-1 macrophages (Riendeau and Kornfeld, 2003). After PMA-differentiation of monocytes into macrophages, THP-1 cells were incubated with *M. smegmatis* for 2 h at multiplicity of infection (MOI) of 25. Extracellular bacteria were then removed and infected cells were treated for 1 h 30 min with lysosomal inhibitor, Bafilomycin A1 (BafA1), or a vehicle control. Autophagy levels were analyzed by LC3-II immunoblotting assay with β-actin as a loading control (Mizushima et al., 2010). Figures 1A,B show that LC3-II/actin ratio increases significantly, by a factor of three, upon *M. smegmatis* infection. To distinguish between induction of LC3-II formation and inhibition of LC3-II lysosomal turn-over, experiments with BafA1 were carried out. Addition of BafA1 increases both LC3-II/actin ratios in non-infected and *M. smegmatis*-infected cells, with LC3-II/actin ratio remaining two times higher in infected cells (Figures 1A,B). These results indicate that basal and functional autophagy is present in THP-1 macrophages and that *M. smegmatis* triggers an upregulation of this process upon infection. Furthermore, we observe that *M. smegmatis*-induced autophagy is maintained at 24 h post-infection and that autophagy levels dependent on MOI (Figure 1C).

Autophagy is known to be regulated at both post-translational and transcriptional levels (Pietrocola et al., 2013). Since *M. smegmatis*-induced autophagy is still observed several hours post-infection, we asked whether *M. smegmatis* infection could trigger expression of autophagy-related genes. Using quantitative real-time RT-PCR, we demonstrate that genes encoding for Atg5, LC3B, hVps34, UVRAG, and STX17 are significantly up-regulated 6 h post-infection (Figure 1D). These data indicate that *M. smegmatis* promotes autophagy, in part, via upregulation of several genes involved in autophagic pathway. In conclusion, as in murine macrophages (Zullo and Lee, 2012), *M. smegmatis* induces a potent autophagic response in human THP-1 macrophages.

### *M. smegmatis* resides in LC3-positive and acidified compartment

**Figure 2:**
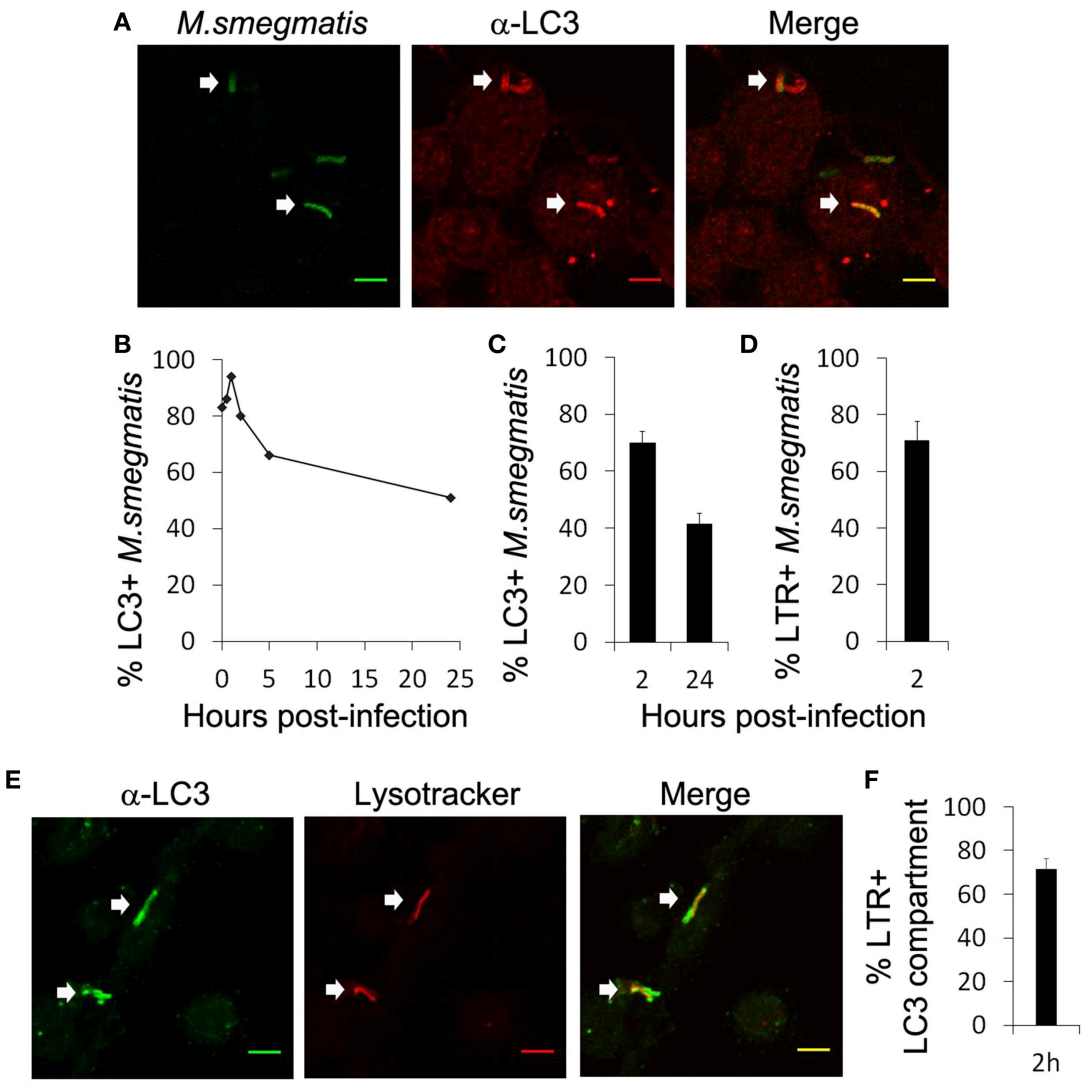
*****M. smegmatis*** resides in LC3-positive and acidified compartment. (A–C)** Differentiated THP-1 were pulsed 30 min with Alexa-488 labeled *M. smegmatis* (*Msm*) at MOI 10, washed, then incubated in THP-1 media for indicated times (hours post-infection). Infected cells were fixed, permeabilized, incubated with antibody against endogenous LC3 and then stained with Alexa-568-labeled secondary antibody. Specimens were analyzed by confocal fluorescence microscopy. **(A)** Representative confocal images of differentiated THP-1 infected with Alexa-488 labeled *M. smegmatis* (green channel) at 2 h post-infection and stained for endogenous LC3 (red channel). Scale bars, 5 μm. **(B)** Kinetic of LC3 association with *M. smegmatis* compartment. **(C)** Quantification of percentage of *M. smegmatis* compartments colocalizing with LC3 at 2 and 24 h post-infection. Data, mean ± s.e.m (*n* = 3 independent experiments). **(D)** Quantification of percentage of *M. smegmatis* compartments colocalizing with LysoTracker Red. Differentiated THP-1 were pulsed 30 min with Alexa-488 labeled *M. smegmatis* at MOI 10, washed, then incubated for 2 h in presence of LysoTracker Red (LTR). Infected cells were washed, fixed, and specimens were analyzed by confocal fluorescence microscopy. Data, mean ± s.e.m (*n* = 3 independent experiments). **(E)** Representative confocal images of differentiated THP-1 infected with *M. smegmatis* stained with LTR and for endogenous LC3. Differentiated THP-1 were pulsed 30 min with *M. smegmatis* at MOI 10, washed, and then chased for 2 h in presence of LysoTracker Red (LTR). Infected cells were fixed, permeabilized, incubated with antibody against endogenous LC3 and then stained with Alexa-568-labeled secondary antibody. Specimens were analyzed by confocal fluorescence microscopy. Scale bars, 5 μm. White arrows indicate colocalization. **(F)** Quantification of percentage of LC3 compartments colocalizing with lysotracker (LTR) at 2 h. Data, mean ± s.e.m (*n* = 3 independent experiments).

Lee's work and these studies clearly demonstrate that *M. smegmatis* enhances autophagy in macrophages (Zullo and Lee, 2012; Figure 1), however, it is unknown whether *M. smegmatis* is targeted by the autophagy machinery. First, we examined endogenous LC3 association with *M. smegmatis* intracellular compartment by immunofluorescence and confocal microscopy (Figure 2A). THP-1 macrophages were pulsed with Alexa 488-labeled *M. smegmatis* for 30 min, incubated for different time points and stained with a specific antibody against LC3. The kinetic of LC3 colocalization with *M. smegmatis* indicates a rapid and robust association of LC3 with mycobacterial compartment shortly after phagocytosis (30 min post-infection) which reaches a maximum around 1 h (Figure 2B). Although LC3 staining diminishes with time, after 24 h we still observe around 50% of *M. smegmatis* associated with LC3 which correlates with LC3-II immunoblots (Figure 1C). LC3 associates with 70% of *M. smegmatis* at 2 h and with 40% at 24 h post-infection (Figure 2C).

Next we asked whether *M. smegmatis* was present in an acidified compartment using Lysotracker tracker red (LTR) staining. We observed that 70% of *M. smegmatis* were already acidified at 2 h post-infection (Figure 2D). Co-staining with an antibody against LC3 shows that around 70% of LC3 compartments were acidified at 2 h which indicates that autophagy process is complete with fusion with late endosomes/lysosomes (Figures 2E,F). To confirm that LC3 compartment containing *M. smegmatis* acquires lysosomal features, we investigated additional markers such as CD63 and DQ Red BSA (Figure S1). DQ Red BSA is a self-quenched red BODIPY dye conjugated to BSA that fluoresces upon enzymatic cleavage by lysosomal proteases. Figure S1 shows that GFP-expressing *M. smegmatis*, found in a LC3 positive compartment, acquires CD63 and DQ Red BSA, which indicates fusion of lysosomes with this compartment. Overall our results indicate that *M. smegmatis* is preferentially targeted by LC3 in macrophages and traffics to a compartment with lysosomal properties.

### TLR2 participates in autophagy activation

**Figure 3:**
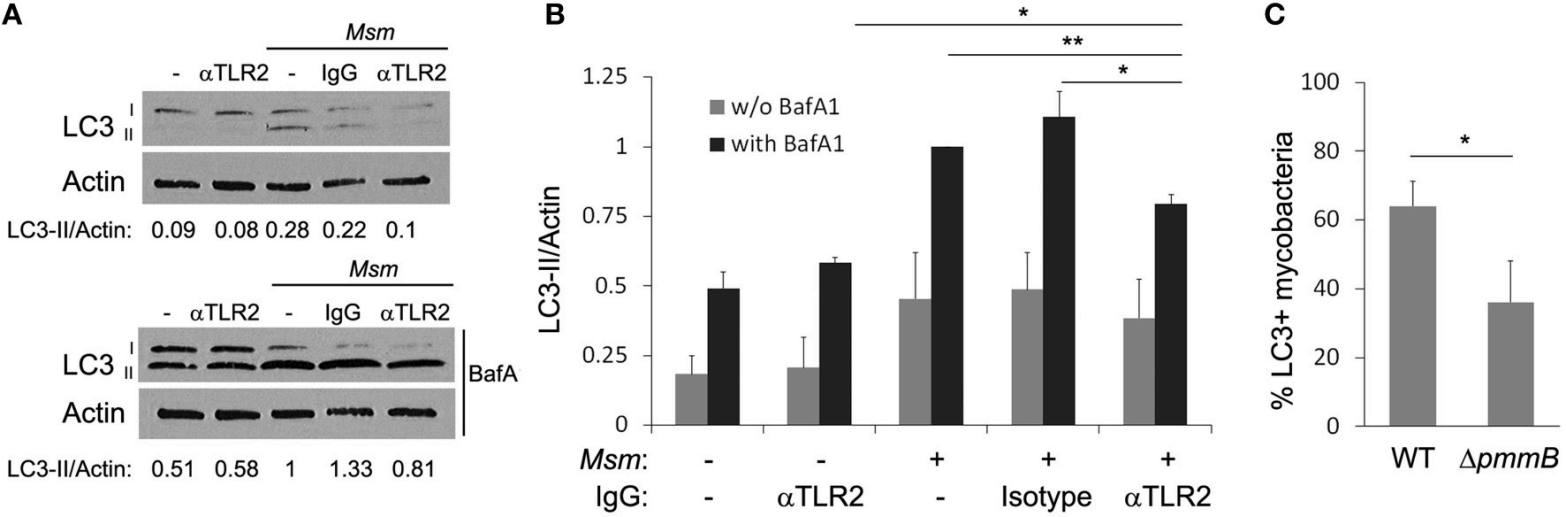
**TLR2 participates in autophagy induction. (A,B)** TLR2 neutralizing antibody reduces LC3-II formation in infected cells. Differentiated THP-1 were pre-incubated for 30 min with IgG (anti-TLR2 or isotype control) at 6 μg/ml, then *M. smegmatis* (*Msm*) was added at MOI 25. After 2 h incubation, cells were washed, and then treated for 1 h and 30 min with 100 nM Bafilomycin A1 (with BafA1) or DMSO control (w/o BafA1). Cells were lysed and analyzed by immunoblotting with anti-LC3 or anti-actin. Densitometric LC3-II/actin ratios are shown underneath the blot **(A)**. LC3-II/actin ratios were measured and normalized to ratio of infected cells with BafA1 **(B)**. Data, mean ± SEM (*n* = 4 independent experiments), ^*^P < 0.05, ^**^P < 0.01, (paired *t*-test). **(C)** Reduced LC3 colocalization with *M. smegmatis* mutant deficient in lipoglycans, TLR2 ligands. Differentiated THP-1 were pulsed 30 min with Alexa-488 labeled *M. smegmatis* wild-type (WT) or Δ*pmmB* at MOI 10, washed, then chased for 2 h. Infected cells were fixed, permeabilized, and stained for endogenous LC3 (as in Figure 2A). Quantification of percentage of *M. smegmatis* compartments colocalizing with LC3 was determined by confocal fluorescence microscopy. Data, mean ± s.e.m (*n* = 3 independent experiments). ^*^P < 0.05 (paired *t*-test).

A mechanism for autophagy activation and LC3 recruitment to bacterial intracellular compartment relies on engagement of PPRs upon phagocytosis (Anand et al., 2011; Mehta et al., 2014). Since TLR2 plays a central role in *M. smegmatis* recognition by THP-1 macrophages (Krishna et al., 2011), its involvement in autophagy induction was investigated. THP-1 macrophages were pre-incubated for 30 min with TLR2 blocking antibody or IgG isotype control prior *M. smegmatis* infection and autophagy level was analyzed by LC3 immunoblotting as in Figures 1A,B. Inhibition of TLR2 reduces significantly, by around 20%, the amount of LC3-II formed (with BafA1) in *M. smegmatis*-infected macrophages (Figures 3A,B). Importantly, no change in LC3-II is observed with isotype control in infected cells, indicating that reduced LC3-II is due to inhibition of *M. smegmatis* recognition by TLR2 and not to non-specific antibody effect *per se*. Furthermore, TLR2 blocking antibody does not alter LC3-II level in non-infected cells demonstrating that the antibody acts on *M. smegmatis*-induced and not on basal autophagy. However, TLR2 blocking antibody does not modify significantly LC3-II steady-state (without BafA1) which indicates that TLR2 activates autophagy by a process that tightly couples LC3-II formation and its turn-over (Figure 3B; Mizushima and Yoshimori, 2007).

Several mycobacterial pathogen-associated molecular patterns (PAMPs) are recognized by TLR2, among them are lipoglycans (Krishna et al., 2011; Basu et al., 2012). A recent study reported that *M. smegmatis* mutant deficient in lipoglycans, Δ*pmmB*, was less able to engage TLR2 which resulted in reduced THP-1 macrophage activation (Krishna et al., 2011). To confirm our result on the role of TLR2-mediated recognition in *M. smegmatis*-induced autophagy, we investigated LC3 association with intracellular compartment containing *M. smegmatis* Δ*pmmB* by immunofluorescence confocal microscopy (Figure S2A). Figure 3C shows that *M. smegmatis* deficient in lipoglycans display significantly less LC3 on its compartment compared to *M. smegmatis* wild-type (around 40% difference). Taken together, these experiments demonstrate that recognition by TLR2 contributes to *M. smegmatis*-induced autophagy, however, since the inhibition was only partial it is most likely that other ligands and/or receptors participate in autophagy induction.

Previous work has shown that NADPH oxidase-generated reactive oxygen species (ROS) are required for TLR2-induced recruitment of LC3 to latex bead-containing phagosome in macrophages (Huang et al., 2009). In our experiments, treatment of THP-1 macrophages with diphenyleneiodonium (DPI), an inhibitor of NADPH oxidase, did not inhibit LC3 association with *M. smegmatis* compartment, as seen by immunofluorescence confocal microcopy, indicating that ROS is not implicated in that process (Figure S2B).

### *M. smegmatis* is not targeted by ubiquitin and autophagy receptors

**Figure 4:**
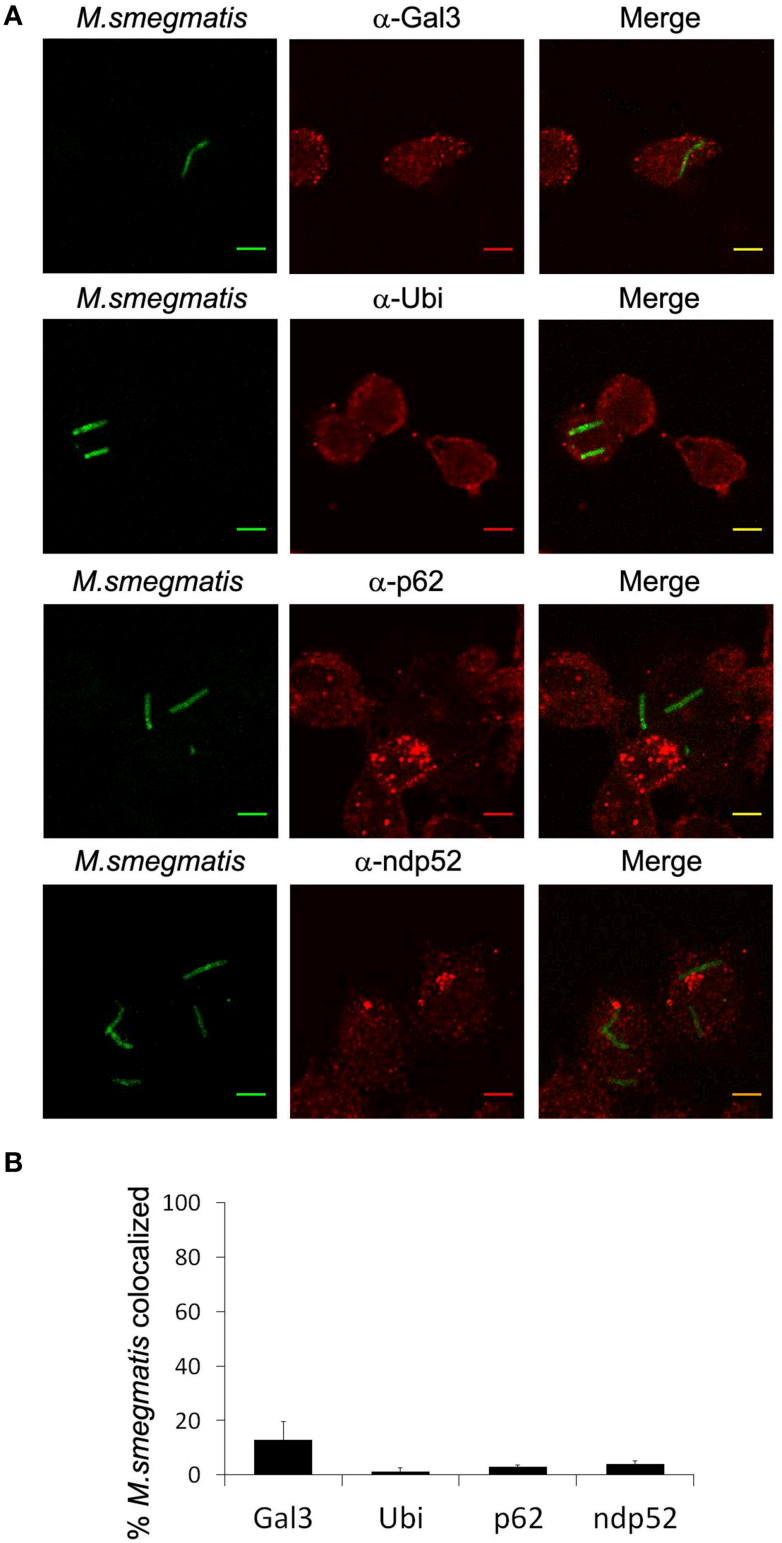
*****M. smegmatis*** is not targeted by ubiquitin and autophagy receptors**. Differentiated THP-1 were pulsed 30 min with Alexa-488 labeled *M. smegmatis* at MOI 10, washed, then incubated in THP-1 media for 2 h. Infected cells were fixed, permeabilized, incubated with antibody against endogenous, Galectin-3, Ubiquitin, p62, or ndp52, and then stained with Alexa-568-labeled secondary antibody. Specimens were analyzed by confocal fluorescence microscopy. **(A)** Representative confocal images of differentiated THP-1 infected with Alexa-488 labeled *M. smegmatis* (green channel) and stained for endogenous Galectin-3 (Gal-3), Ubiquitin (Ubi), p62, or ndp52 (red channel). Scale bars, 5 μm. **(B)** Quantification of percentage of *M. smegmatis* compartments colocalizing with Galectin-3 (Gal-3), Ubiquitin (Ubi), p62 or ndp52. Data, mean ± s.e.m (*n* = 3 independent experiments).

LC3 was shown to be associated with *M. tuberculosis* compartment via an ubiquitin- and p62-dependent mechanism (Watson et al., 2012). Esx-1 secretion system of *M. tuberculosis* damages phagosomal membrane which triggers ubiquitination and recruitment of autophagy receptors such as p62 and ndp52, on mycobacterial compartment. Although *M. smegmatis* does not escape into the cytosol and its Esx-1 system appears not to possess, *in vitro*, membrane-lytic properties (De Leon et al., 2012; Houben et al., 2012; Simeone et al., 2012), we wondered whether LC3 could target *M. smegmatis* via a molecular mechanism similar to the one observed with *M. tuberculosis*. First, we confirmed that *M. smegmatis* compartment was not damaged using immunofluorescence confocal microscopy and staining of endogenous Galectin-3, a marker for phagosomal membrane rupture (Paz et al., 2010). Less than 15% of *M. smegmatis* colocalizes with Galectin-3 (Figures 4A,B). Next, we investigated ubiquitin association with *M. smegmatis* using an antibody recognizing mono- and polyubiquitinylated conjugates. Figures 4A,B show that <2% of bacteria colocalize with ubiquitin at 2 h post-infection whereas more than 60% colocalize with LC3 (Figure 3B), suggesting that LC3 is recruited in an ubiquitin independent-manner. To validate this conclusion, we sought to examine two autophagy receptors known to bind ubiquitin and LC3. As for ubiquitin, both p62 and ndp52 are absent from *M. smegmatis* compartment (Figures 4A,B). Importantly, we could detect Galectin-3, ubiquitin, p62, and ndp52 colocalization with GFP-*M. tuberculosis* which validates the use of these antibodies in THP-1 cells (Figure S3). Altogether, these data demonstrate that LC3 targeting of *M. smegmatis* is not triggered by membrane damage and does not involve ubiquitin labeling and classical autophagy receptors.

### Autophagy-related proteins targets *M. smegmatis* to promote killing

**Figure 5:**
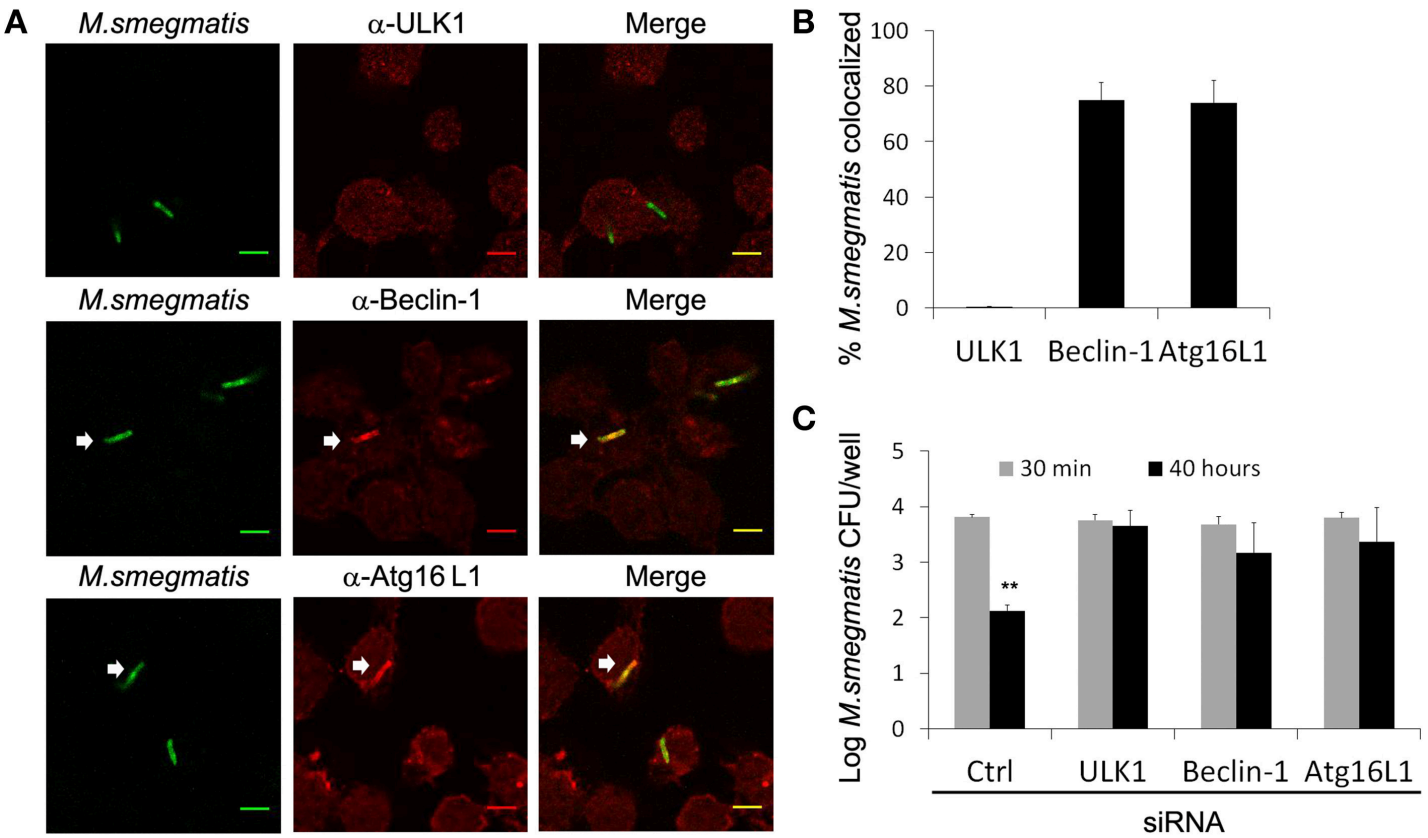
**Autophagy-related proteins target ***M. smegmatis*** to promote killing. (A,B)** Differentiated THP-1 were pulsed 30 min with Alexa-488 labeled *M. smegmatis* at MOI 10, washed, then incubated in THP-1 media for 2 h. Infected cells were fixed, permeabilized, incubated with antibody against endogenous ULK-1, Beclin-1, or Atg16L1 and then stained with Alexa-568-labeled secondary antibody. Specimens were analyzed by confocal fluorescence microscopy. **(A)** Representative confocal images of differentiated THP-1 infected with Alexa-488 labeled *M. smegmatis* and stained for endogenous ULK1, Beclin-1, or Atg16L1. Scale bars, 5 μm. **(B)** Quantification of percentage of *M. smegmatis* compartments colocalizing with ULK1, Beclin-1, and Atg16L1. Data, mean ± s.e.m (*n* = 3 independent experiments). **(C)** *M. smegmatis* killing by macrophages is reduced after ULK1, Beclin-1, or Atg16L1 knockdown. THP-1 were transfected with control siRNA (Ctrl) or specific siRNA for 24 h in presence of PMA, washed, then infected with *M. smegmatis* at MOI 2. After 30 min infection, cells were washed, incubated in THP-1 media for 30 min and, then, either directly lysed for uptake measurement or lysed after 40 h incubation. Bacteria were plated on 7H11 agar plates and CFU were counted after 3 days. Graph represents log CFU after uptake (30 min) and after 40 h post-infection. Data, mean ± SEM (*n* = 3 independent experiments), ^**^P < 0.01 (paired *t*-test). White arrows indicate colocalization.

Although LC3 is used as a gold standard autophagic marker, recent reports pointed out that LC3 can also be involved in non-canonical autophagy and autophagy-independent processes (Codogno et al., 2012; Bestebroer et al., 2013). To confirm that autophagy machinery targets *M. smegmatis* in macrophages, we examined intracellular localization of endogenous ULK1, Beclin-1, and Atg16L1, three early Atgs from distinct functional groups, involved in autophagosome formation upstream of LC3 recruitment (Carlsson and Simonsen, 2015). Immunofluorescence confocal microscopy analysis and quantification show that ~80% of mycobacteria are decorated with Beclin-1 and Atg16L1 but not with ULK1 at 2 h post-infection (Figures 5A,B). Kinetic of Atg13 recruitment confirm that ULK1 complex does not accumulate on *M. smegmatis* compartments (Figures S4B,C). Importantly, control experiment indicates that ULK1 and Atg13 antibodies are functional as we observed punctate structures ULK1- and p62- positives (Figure S5) and Atg13- and p62- positives (Figure S4A) upon cell starvation. However, we cannot rule out the possibility that association of ULK1 complex with *M. smegmatis* compartment is low and/or short-lived.

After reaching an acidified compartment, *M. smegmatis* is known to be killed by macrophages, however, whether Atgs are involved in that process remained to be investigated (Gutierrez et al., 2008). Since some Atgs may have autophagy-independent functions, it is highly recommended to investigate several Atgs implicated in different protein complexes and autophagy steps (Klionsky et al., 2016). Thus, THP-1 were transfected with siRNA against ULK1, Beclin-1, and Atg16L1, or control siRNA for 24 h, then infected with *M. smegmatis*. After 30 min and 40 h post-infection, macrophages were lysed and number of bacteria was determined by colony-forming unit (CFU). As expected we observe a significant killing of *M. smegmatis* by macrophages transfected with control siRNA, with a reduction of 2 log CFU after 2 days (Figure 5C). In contrast, no significant decrease of CFU was observed in macrophages depleted for ULK1, Beclin-1, or Atg16L1 indicating that Atgs are required to control *M. smegmatis* infection (Figure 5C; Figure S6). Importantly, knockdowns of Atgs did not alter phagocytic capacity of macrophages as we observe an identical number of CFU at 30 min post-infection in control and Atg-depleted cells (Figure 5C). In conclusion these results demonstrate that autophagy participates in innate immune defense against *M. smegmatis.*

## Discussion

Although, several studies have reported that autophagy activation is an innate immune response of macrophages to mycobacteria, the underlying molecular mechanisms and the role of Atgs in this response remained to be fully characterized (Lerena and Colombo, 2011; Watson et al., 2012; Zullo and Lee, 2012; Wang et al., 2013). In this work, we demonstrate for the first time that non-pathogenic *M. smegmatis* stimulates autophagy through TLR2 engagement. Furthermore, *M. smegmatis* regulates autophagy at a transcriptional level by triggering expression of several autophagy-related genes. We found that Atgs targeting of *M. smegmatis* does not rely on ubiquitin-coating or autophagy receptor recruitment. Thus, according to the species, mycobacteria are recognized by the autophagic machinery via, at least two mechanisms: one independent of ubiquitin (our study) and the other dependent of ubiquitin (Watson et al., 2012). Importantly, we demonstrate that autophagy machinery mobilization endows macrophages with enhanced bactericidal properties against *M. smegmatis*.

TLR2 is known to participate in autophagy activation upon *Listeria monocytogenes* and *Staphylococcus aureus* infections in murine macrophages (Anand et al., 2011; Fang et al., 2014). However, the type of autophagy triggered by these bacteria was not investigated A role for mycobacterial recognition by TLRs in autophagy activation was recently unveiled in zebrafish and human macrophages (van der Vaart et al., 2014). Authors found that TLRs engagement triggers expression of DRAM1 which mediates selective autophagy of *M. marinum* and *M. tuberculosis* via a STING/Ubiquitin/p62 pathway. Several purified TLR2 ligands, including mycobacterial compounds, are known to trigger autophagy (Shin et al., 2010). Here, our study demonstrates for the first time that TLR2 engagement by the whole live mycobacteria, *M. smegmatis*, can trigger autophagy independently of ubiquitin and p62 indicating that multiple autophagic pathways may be regulated by TLRs upon mycobacterial infections. Here, we found that, in contrast to *M. tuberculosis, M. smegmatis* is not ubiquitinated and does not recruit autophagy receptor proteins. Although not completely unexpected, this result supports, *in vitro*, study showing the incapacity of *M. smegmatis* Esx-1 effector to damage membrane (De Leon et al., 2012). Finally, *M. smegmatis* can also be recognized by NOD2 and Dectin-1, both known to trigger targeting of bacteria by autophagy machinery, thus, it is most likely that other ubiquitin-independent mechanisms are at play in this process (Yadav and Schorey, 2006; Coulombe et al., 2009; Travassos et al., 2010; Ma et al., 2012).

LC3 associates with *M. smegmatis* compartment at early time point post-infection suggesting a link between phagocytosis and autophagy machinery recruitment. Several Atg-dependent pathways involved in clearance of phagocytosed material have been described. The first discovered was LC3-associated phagocytosis, also called LAP (Sanjuan et al., 2007). This process which relies on LC3 conjugation directly onto the phagosomal membrane requires ROS production and most of autophagy machinery except ULK1 complex (Mehta et al., 2014). LAP can accelerate or delay degradation of engulfed material depending on the cellular context (Munz, 2014). However, additional pathways linking Atgs and phagocytosis have been uncovered which depend on ULK1. In *Caenorhabditis elegans*, apoptotic corpse clearance requires all the Atgs including ULK1 and Atg13 (Cheng et al., 2013). More recently, a study has shown that in epithelial cells, phagosome containing apoptotic cell and decorated with ubiquitin is engulfed into an autophagosome via an ULK1-dependent pathway (Brooks et al., 2015). *M. smegmatis* is found in a single membrane-bound vacuole (Frehel et al., 1986; Houben et al., 2012) and is not ubiquitinated (Figure 4) indicating that the phagosome is not sequestered into an autophagosome. Furthermore, even thought we did not observe ULK1 on *M. smegmatis* phagosome, we found a role for ULK1 in bacterial killing suggesting a pathway different from LC3-associated phagocytosis. Finally, we cannot rule out the possibility that part of LC3 targeting results from fusion of autophagosomes or autophagic precursors with *M. smegmatis*-containing phagosome. Importantly, this autophagic innate immune response is essential for the killing of mycobacteria enclosed in a damage-free phagosome.

To our knowledge, this is the first study to show that *M. smegmatis* promotes expression of several genes encoding autophagy-related proteins. Gutierrez and colleagues have reported that *M. smegmatis* upregulates expression of numerous genes encoding for lysosomal and membrane trafficking proteins, though, none of belonged to the autophagy machinery (Gutierrez et al., 2008). More recently, mycobacteria-induced NFkappaB activation was shown to promote expression of *dram1*, an effector of STING/p62-mediated autophagy (van der Vaart et al., 2014). Here, *M. smegmatis*-induced expression of *STX17* and *LC3B* is not regulated by NFkappaB (data not shown). Several transcription factors controlling *LC3B* expression have been identified depending on the biological context, however, nothing is known about those regulating *STX17* expression (Pietrocola et al., 2013; Fullgrabe et al., 2014). Thus, it would be of great interest to determine which transcription factors are involved in *M. smegmatis*-induced expression of autophagy-related genes. Additionally, *M. smegmatis* downregulates or upregulates expression of several microRNAs in macrophages (Bettencourt et al., 2013). Since several autophagy genes can be targeted by microRNAs (Fullgrabe et al., 2014), one can hypothesize that *M. smegmatis* may also regulate Atgs at a post-transcriptional level.

To summarize, we have uncovered an alternative autophagic pathway targeting mycobacteria which relies, in part, on TLR2 engagement but not on membrane damage and ubiquitin-coating. This finding is particularly relevant for our understanding of autophagic response in innate immunity against mycobacteria. Improved knowledge of this process may help in designing more efficient vaccine and host-based therapeutic approaches against mycobacterial infections.

## Author contributions

AB, CL, IV participated in design of study. AB, CL, IV participated in execution of study and analysis of samples and data. IV wrote the manuscript. AB revised the manuscript.

### Conflict of interest statement

The authors declare that the research was conducted in the absence of any commercial or financial relationships that could be construed as a potential conflict of interest.

## Supplementary material

Figure S1 ***M. smegmatis*-containing LC3-positive compartment acquires CD63, DQ Red BSA, and lysotracker**. Differentiated THP-1 were pulsed 30 min with GFP-expressing ***M. smegmatis*** at MOI 10, washed, then incubated for 2 h in absence **(A,B)** or presence of lysoTracker Red (LTR) **(C)**. For DQ Red BSA assay, cells were preincubated for 2 h with 10 μg/ml DQ Red BSA before infection **(B)**. Infected cells were fixed, permeabilized, incubated with rabbit antibody against endogenous LC3 and then stained with Alexa-647-labeled secondary antibody. **(A)** Cells were incubated with rabbit antibody against endogenous LC3 and mouse antibody against endogenous CD63 followed by staining with Alexa-647-labeled and Alexa-568-labeled secondary antibodies, respectively. Specimens were analyzed by confocal fluorescence microscopy. Confocal images show LC3 compartment (cyan channel) containing GFP-expressing *M. smegmatis* (green channel) colocalizing with CD63, DQ Red BSA, or LTR (red channel). Scale bars, 5 μm. White arrows indicate colocalization.

Figure S2 **LC3 colocalization with ***M. smegmatis*** compartment. (A)** Representative confocal images of differentiated THP-1 infected with Alexa-488 labeled *M. smegmatis* Δ*pmmB* at MOI 10 (green channel) at 2 h post-infection and stained for endogenous LC3 (red channel). Scale bars, 5 μm. **(B)** Inhibition of reactive oxygen species does not impair LC3 association with *M. smegmatis* compartment. Differentiated THP-1 were pre-incubated or not for 30 min with diphenyleneiodonium (DPI) at 20 μM, then for pulsed 30 min with Alexa-488 labeled *M. smegmatis* at MOI 10, washed, then chased for 2 h in presence or absence of DPI. Infected cells were fixed, permeabilized, and stained for endogenous LC3. Quantification of percentage of *M. smegmatis* compartments colocalizing with LC3 was determined by confocal fluorescence microscopy. Data, mean ± s.e.m (*n* = 3 independent experiments).

Figure S3 *****M. tuberculosis*** is targeted by ubiquitin and autophagy receptors in THP-1 macrophages**. Differentiated THP-1 were pulsed 1 h with GFP-expressing *M. tuberculosis* at MOI 10, washed, then chased for 3 days. Infected cells were fixed, permeabilized, incubated with antibody against endogenous, Galectin-3, Ubiquitin, p62 or ndp52, and then stained with Alexa-568-labeled secondary antibody. Specimens were analyzed by confocal fluorescence microscopy. Confocal images show GFP-expressing *M. tuberculosis* (green channel) colocalizing with endogenous Galectin-3 (Gal-3), Ubiquitin (Ubi), p62 or ndp52 (red channel). Scale bars, 5 μm. White arrows indicate colocalization.

Figure S4 **Validation of Atg13 antibody and kinetic of Atg13 association with ***M. smegmatis*** compartment. (A)** Differentiated THP-1 were starved with EBSS media for 6 h, fixed, permeabilized, incubated with antibody against endogenous p62 (mouse) and Atg13 (rabbit) and then stained with Alexa-488- and Alexa-568-labeled secondary antibody, respectively. Representative confocal images of starved THP-1 stained for endogenous p62 (green channel) and Atg13 (red channel). Scale bars, 2 μm. White arrows indicate colocalization. **(B,C)** Differentiated THP-1 were pulsed 30 min with Alexa-488 labeled *M. smegmatis* (*Msm*) at MOI 10, washed, then incubated in THP-1 media for indicated times (hours post-infection). Infected cells were fixed, permeabilized, incubated with antibody against endogenous Atg13 and then stained with Alexa-568-labeled secondary antibody. Specimens were analyzed by confocal fluorescence microscopy. **(B)** Representative confocal images of differentiated THP-1 infected with Alexa-488 labeled *M. smegmatis* (green channel) at 2 h post-infection and stained for endogenous Atg13 (red channel). Scale bars, 2 μm. **(C)** Kinetic of Atg13 association with *M. smegmatis* compartment.

Figure S5 **Validation of ULK1 antibody**. Differentiated THP-1 were starved with EBSS media for 6 h, fixed, permeabilized, incubated with antibody against endogenous p62 (mouse) and ULK1 (rabbit) and then stained with Alexa-488- and Alexa-568-labeled secondary antibody, respectively. Representative confocal images of starved THP-1 stained for endogenous p62 (green channel) and ULK1 (red channel). Scale bars, 2 μm. White arrows indicate colocalization.

Figure S6 **ULK1, Beclin-1, Atg16L1 knockdown**. The extent of knockdown was analyzed by immunoblotting using antibodies against ULK1, Beclin-1, or Atg16L1. Actin was used as a loading control. Ctrl: control siRNA.
