# Supplementary figures and images for: Autophagy-Related Proteins Target Ubiquitin-Free Mycobacterial Compartment to Promote Killing in Macrophages
Source: Front Cell Infect Microbiol. 2016 May 11;6:53. doi: 10.3389/fcimb.2016.00053 (PMC4863073; doi:10.3389/fcimb.2016.00053)

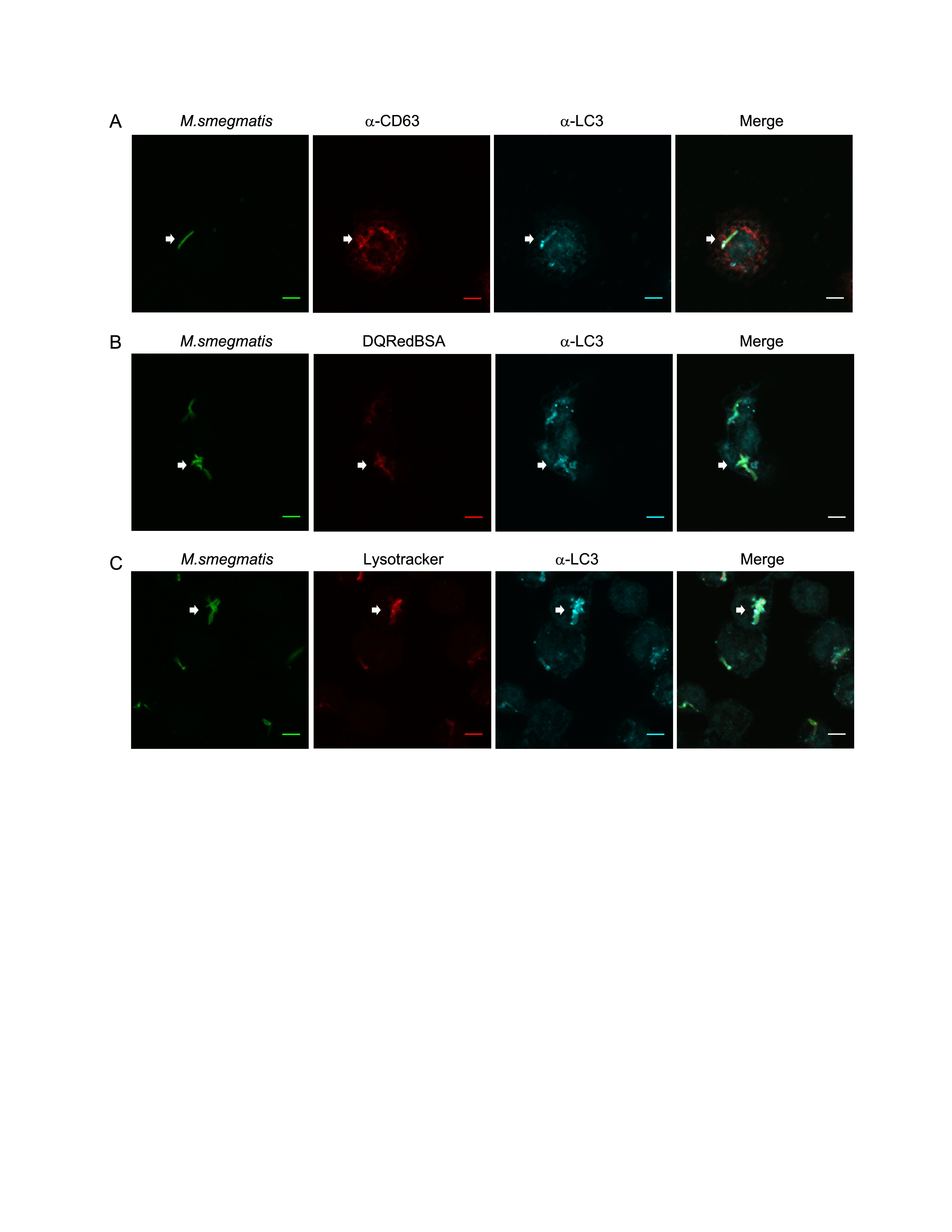

Supplement: Figure S1 — M. smegmatis-containing LC3-positive compartment acquires CD63, DQ Red BSA, and lysotracker. Differentiated THP-1 were pulsed 30 min with GFP-expressing M. smegmatis at MOI 10, washed, then incubated for 2 h in absence (A,B) or presence of lysoTracker Red (LTR) (C). For DQ Red BSA assay, cells were preincubated for 2 h with 10 μg/ml DQ Red BSA before infection (B). Infected cells were fixed, permeabilized, incubated with rabbit antibody against endogenous LC3 and then stained with Alexa-647-labeled secondary antibody. (A) Cells were incubated with rabbit antibody against endogenous LC3 and mouse antibody against endogenous CD63 followed by staining with Alexa-647-labeled and Alexa-568-labeled secondary antibodies, respectively. Specimens were analyzed by confocal fluorescence microscopy. Confocal images show LC3 compartment (cyan channel) containing GFP-expressing M. smegmatis (green channel) colocalizing with CD63, DQ Red BSA, or LTR (red channel). Scale bars, 5 μm. White arrows indicate colocalization. [file Image1.JPEG]

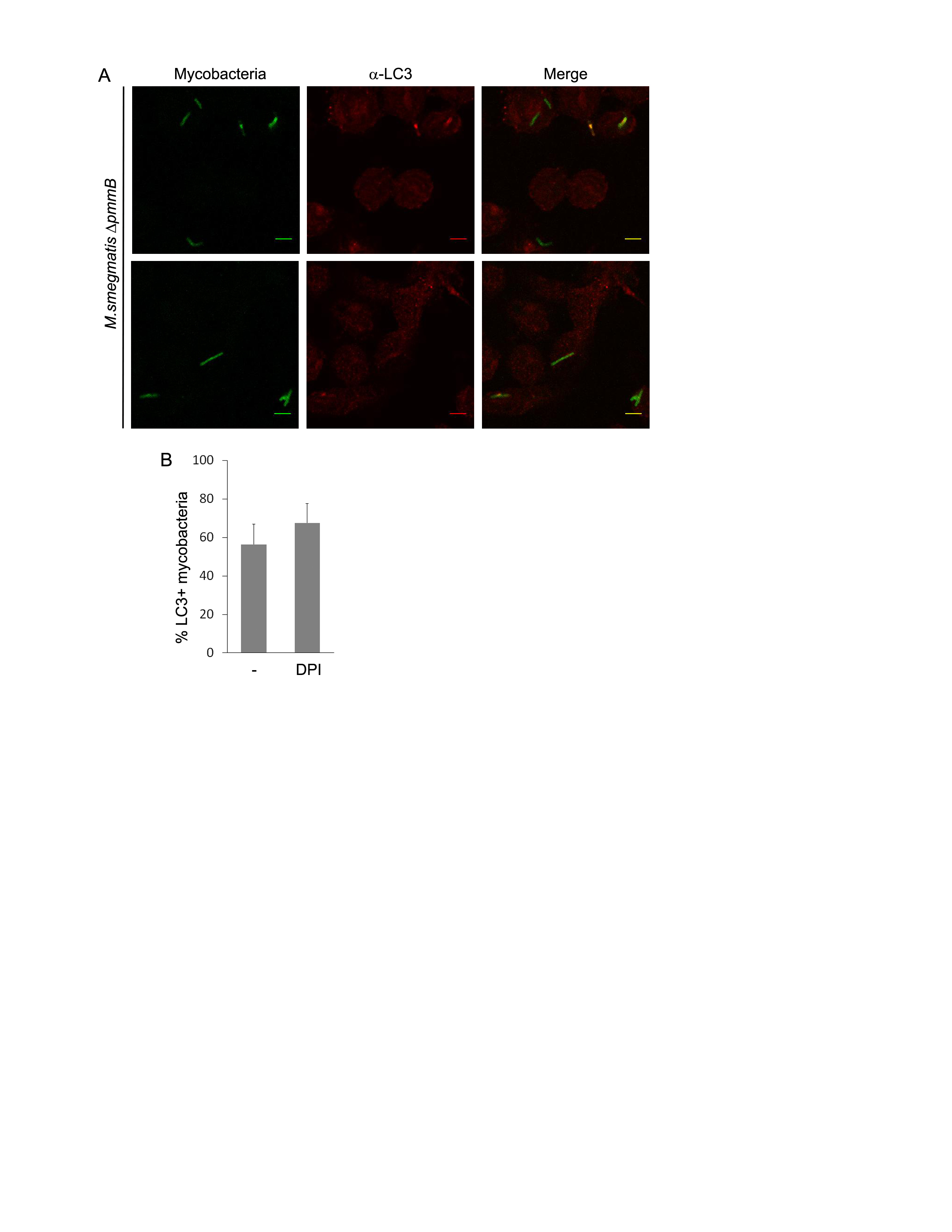

Supplement: Figure S2 — LC3 colocalization with M. smegmatis compartment. (A) Representative confocal images of differentiated THP-1 infected with Alexa-488 labeled M. smegmatis ΔpmmB at MOI 10 (green channel) at 2 h post-infection and stained for endogenous LC3 (red channel). Scale bars, 5 μm. (B) Inhibition of reactive oxygen species does not impair LC3 association with M. smegmatis compartment. Differentiated THP-1 were pre-incubated or not for 30 min with diphenyleneiodonium (DPI) at 20 μM, then for pulsed 30 min with Alexa-488 labeled M. smegmatis at MOI 10, washed, then chased for 2 h in presence or absence of DPI. Infected cells were fixed, permeabilized, and stained for endogenous LC3. Quantification of percentage of M. smegmatis compartments colocalizing with LC3 was determined by confocal fluorescence microscopy. Data, mean ± s.e.m (n = 3 independent experiments). [file Image2.JPEG]

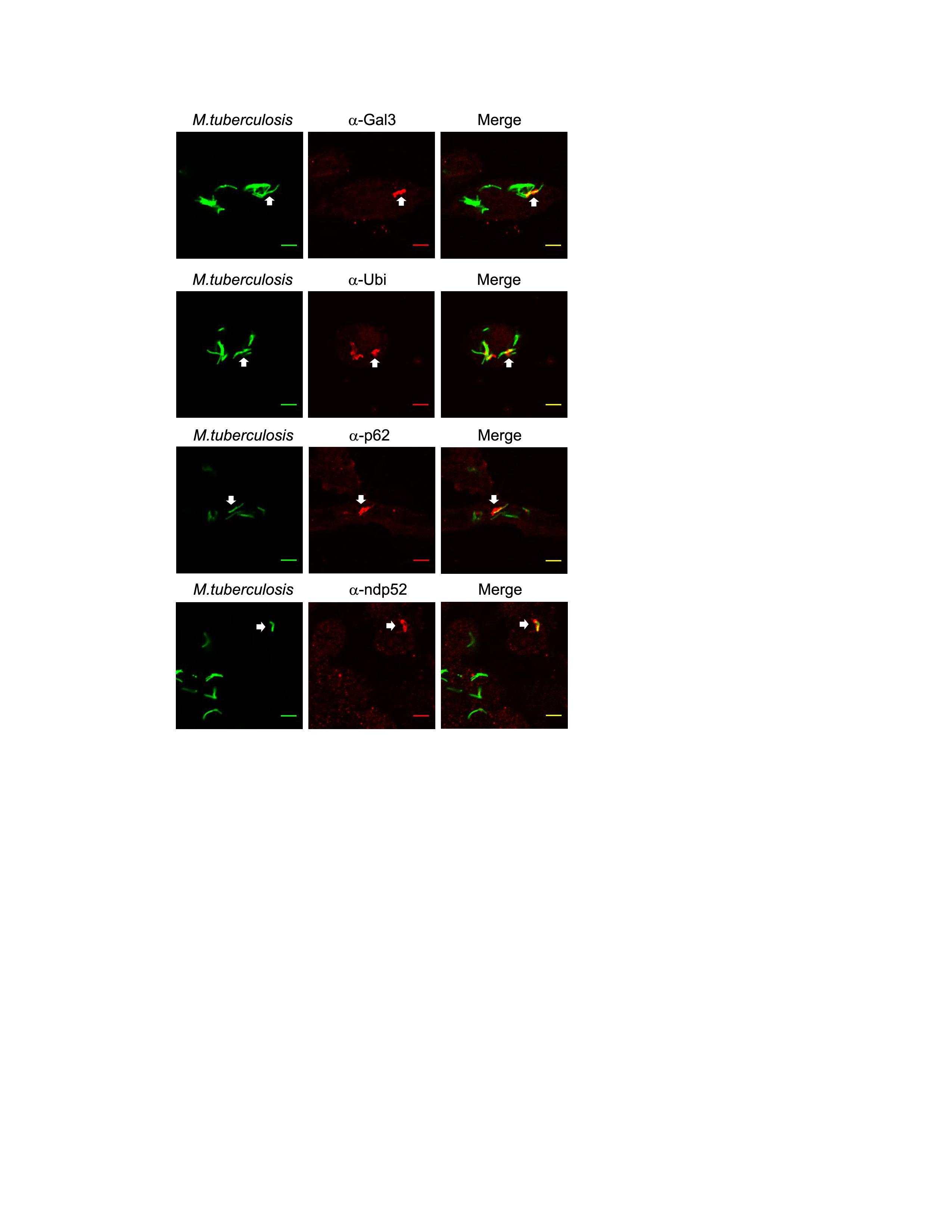

Supplement: Figure S3 — M. tuberculosis is targeted by ubiquitin and autophagy receptors in THP-1 macrophages. Differentiated THP-1 were pulsed 1 h with GFP-expressing M. tuberculosis at MOI 10, washed, then chased for 3 days. Infected cells were fixed, permeabilized, incubated with antibody against endogenous, Galectin-3, Ubiquitin, p62 or ndp52, and then stained with Alexa-568-labeled secondary antibody. Specimens were analyzed by confocal fluorescence microscopy. Confocal images show GFP-expressing M. tuberculosis (green channel) colocalizing with endogenous Galectin-3 (Gal-3), Ubiquitin (Ubi), p62 or ndp52 (red channel). Scale bars, 5 μm. White arrows indicate colocalization. [file Image3.JPEG]

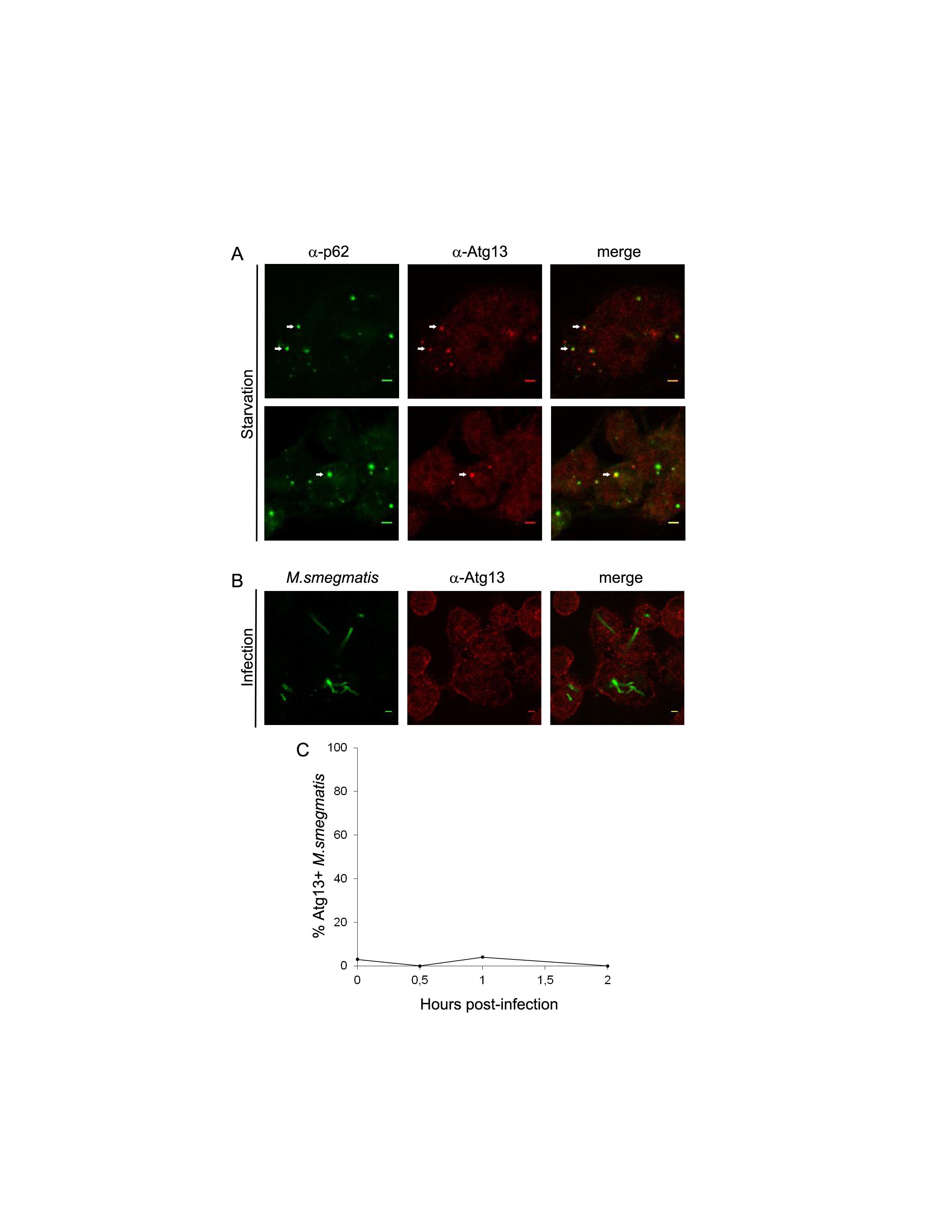

Supplement: Figure S4 — Validation of Atg13 antibody and kinetic of Atg13 association with M. smegmatis compartment. (A) Differentiated THP-1 were starved with EBSS media for 6 h, fixed, permeabilized, incubated with antibody against endogenous p62 (mouse) and Atg13 (rabbit) and then stained with Alexa-488- and Alexa-568-labeled secondary antibody, respectively. Representative confocal images of starved THP-1 stained for endogenous p62 (green channel) and Atg13 (red channel). Scale bars, 2 μm. White arrows indicate colocalization. (B,C) Differentiated THP-1 were pulsed 30 min with Alexa-488 labeled M. smegmatis (Msm) at MOI 10, washed, then incubated in THP-1 media for indicated times (hours post-infection). Infected cells were fixed, permeabilized, incubated with antibody against endogenous Atg13 and then stained with Alexa-568-labeled secondary antibody. Specimens were analyzed by confocal fluorescence microscopy. (B) Representative confocal images of differentiated THP-1 infected with Alexa-488 labeled M. smegmatis (green channel) at 2 h post-infection and stained for endogenous Atg13 (red channel). Scale bars, 2 μm. (C) Kinetic of Atg13 association with M. smegmatis compartment. [file Image4.JPEG]

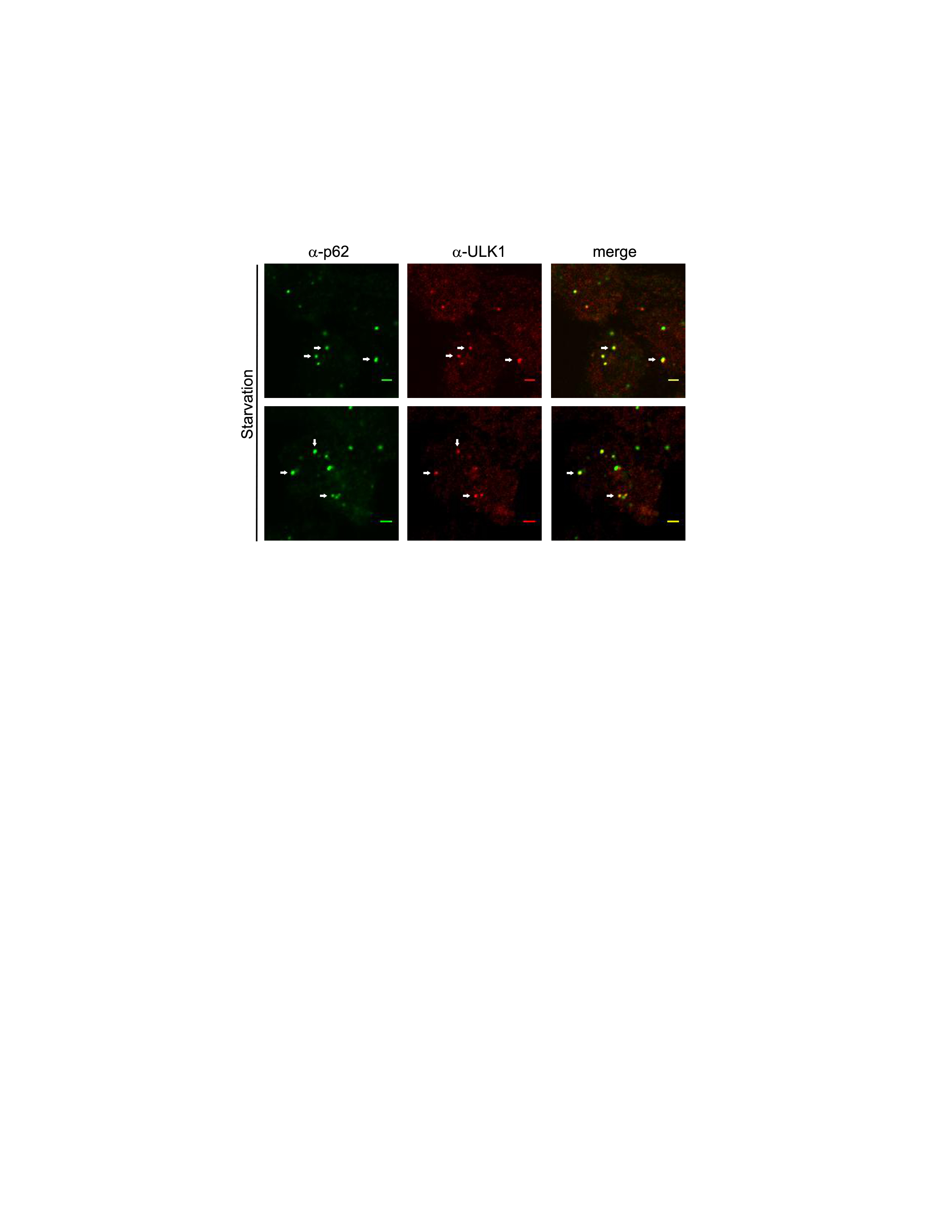

Supplement: Figure S5 — Validation of ULK1 antibody. Differentiated THP-1 were starved with EBSS media for 6 h, fixed, permeabilized, incubated with antibody against endogenous p62 (mouse) and ULK1 (rabbit) and then stained with Alexa-488- and Alexa-568-labeled secondary antibody, respectively. Representative confocal images of starved THP-1 stained for endogenous p62 (green channel) and ULK1 (red channel). Scale bars, 2 μm. White arrows indicate colocalization. [file Image5.JPEG]

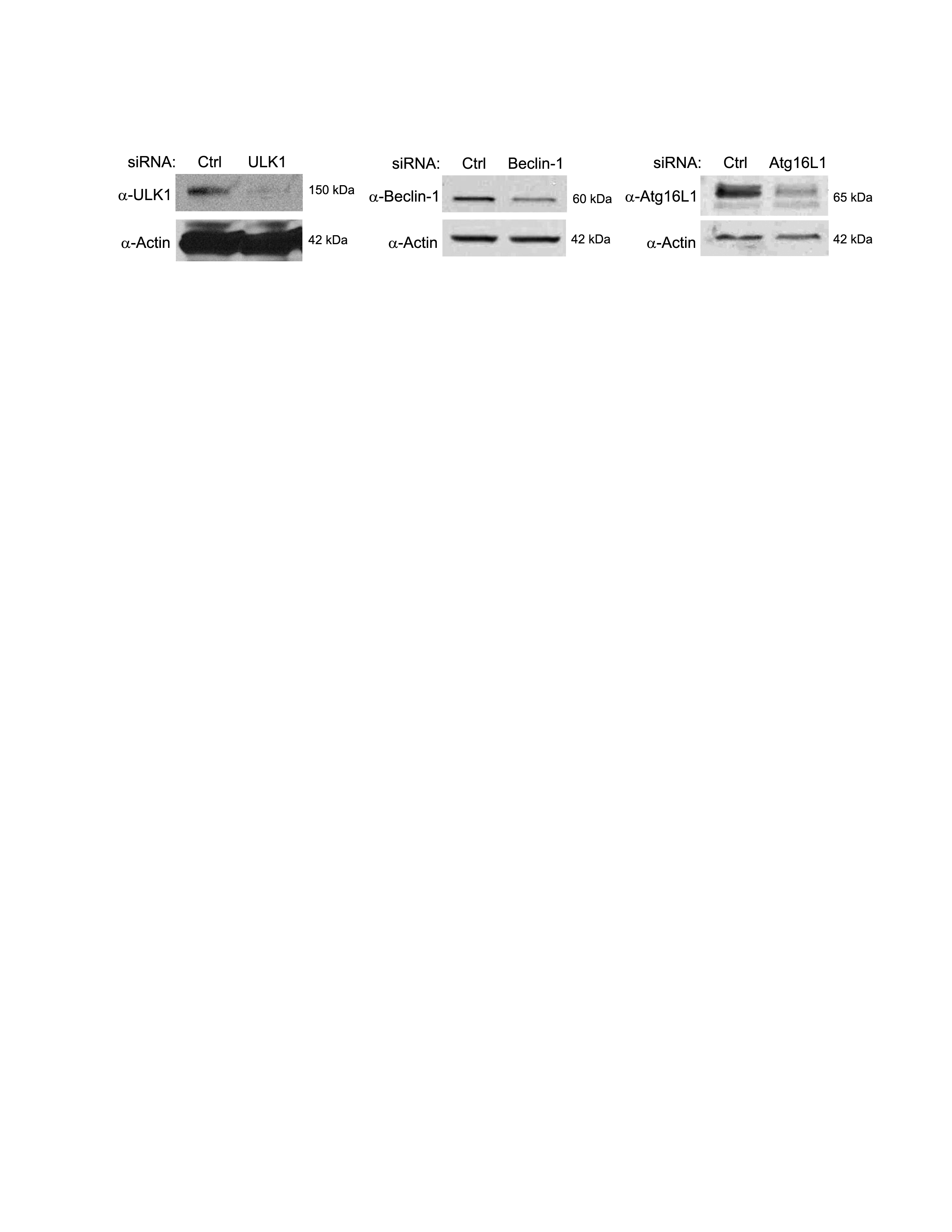

Supplement: Figure S6 — ULK1, Beclin-1, Atg16L1 knockdown. The extent of knockdown was analyzed by immunoblotting using antibodies against ULK1, Beclin-1, or Atg16L1. Actin was used as a loading control. Ctrl: control siRNA. [file Image6.JPEG]
